# Surgical management of dissecting cellulitis of the scalp using free latissimus dorsi flap and meshed split–thickness skin graft: A case report

**DOI:** 10.1097/MD.0000000000024092

**Published:** 2021-01-29

**Authors:** Nicoleta-Sara Baneu, Vlad Adam Bloancă, Diana Szilagyi, Patricia Cristodor, Alexandru Pesecan, Tiberiu Ioan Bratu, Zorin Petrişor Crăiniceanu

**Affiliations:** a “Pius Brînzeu” Emergency County Hospital Timişoara, Department of Plastic and Reconstructive Surgery; b “Victor Babeş” University of Medicine and Pharmacy, Faculty of Medicine; c “Pius Brînzeu” Emergency County Hospital Timişoara, Department of Pathology; d Emergency Clinical Municipal Hospital, Timişoara, Department of Dermatology, Romania.

**Keywords:** case report, dissecting cellulitis of the scalp, free latissimus dorsi flap, Hoffman disease

## Abstract

**Introduction:**

Dissecting cellulitis of the scalp, or Hoffman disease, is described as an extremely rare condition. Clinically, it is represented by recurrent painful nodules, purulent drainage, interconnected sinus tracts and keloid formation, leading to scaring and cicatricial alopecia. Without a precise diagnosis and an adequate treatment, the repercussions consist of severe infectious complications along with psychological negative effects and serious aesthetic alterations. There is no standard treatment. In refractory cases, surgical management is reported.

**Patient concerns:**

We report a case of a 65-year-old Caucasian male patient, with a 5-year history of Hoffman disease, who presented with multiple abscesses and sinus tracts of the scalp and patches of alopecia. The lesions were non-responsive to medical treatment.

**Diagnosis:**

The diagnosis of DCS has been established on the basis of the clinical appearance and has been confirmed histopathologically.

**Interventions:**

The patient underwent wide excision of the scalp, followed by reconstruction using free latissimus dorsi flap and covered by meshed split-thickness skin graft.

**Outcomes:**

Eighteen-month follow-up revealed complete remission of symptoms and lesions along with satisfactory cosmetic result.

**Conclusion:**

The scope of this case report is to raise awareness of the following aspects: Hoffman disease has an extremely low occurrence rate, a difficult differential diagnosis and no standard therapeutical strategy. It also highlights the effectiveness of scalpectomy and free latissimus dorsi flap covered by meshed split-thickness skin graft in treating a very advanced stage of the disease together with providing a natural contouring of the scalp. Ultimately, it discusses the other treatment alternatives.

## 1 Introduction

Dissecting cellulitis of the scalp or Hoffman disease or Perifolliculitis Capitis Abscedens et Suffodiens is described as a rare condition of unknown etiology that affects the scalp, characterized by recurrent pustules, nodules with purulent drainage and interconnected sinus tract formation leading to scarring and alopecia.

It occurs more frequently in African–American men between 18 and 40 years-old. It has no standard treatment, nor an officially accepted classification. Hoffman disease is part of the follicular occlusion tetrad along with acne conglobata, hidradenitis suppurativa, and pilonidal sinus. These 4 disorders share a common pathogenesis, mainly consisting of hyperkeratosis followed by follicular occlusion. The bacterial infection is often found, but appears as a secondary phenomenon and the cultures in the closed abscesses are frequently negative.^[1,2,3]^

### 1.1 Case report

Written informed consent was obtained from the patient.

**Figure 1:**
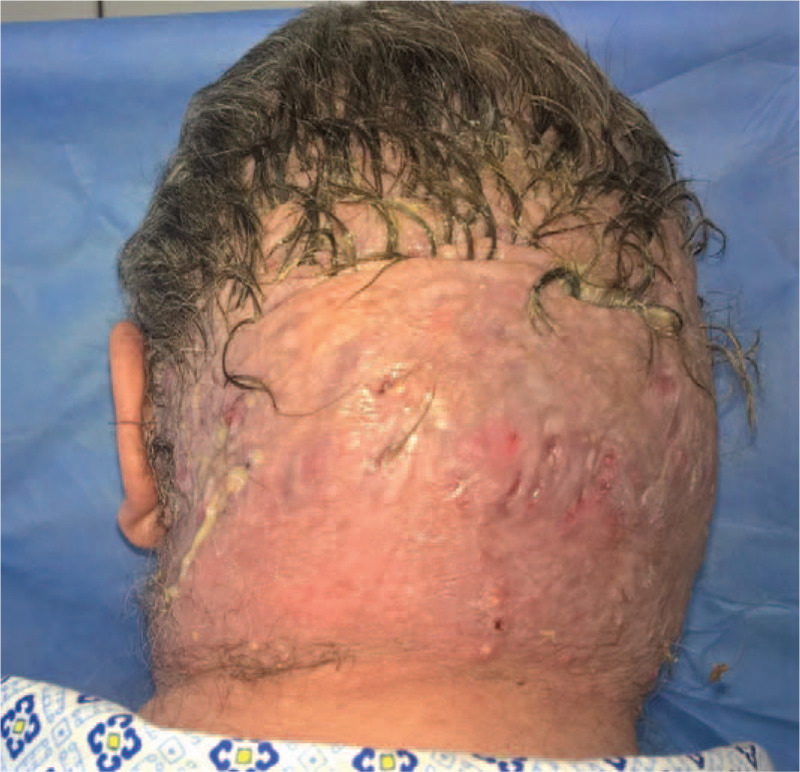
Multiple fluctuant nodules covering most of the scalp.

**Figure 2:**
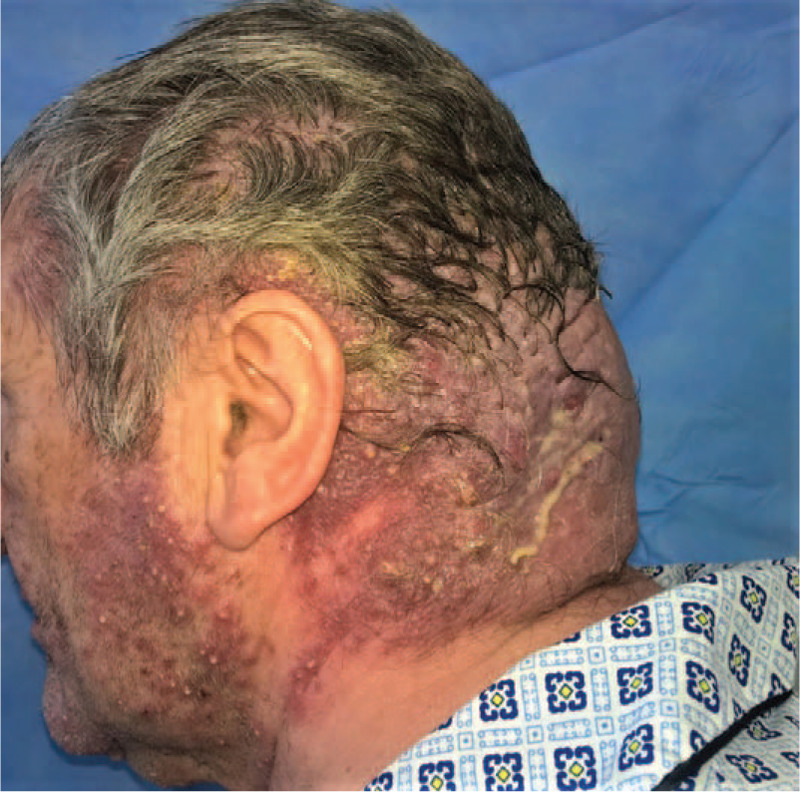
The lesions extend to the posterior cervical region and beard area of the face.

We report a case of a 65-year-old Caucasian male patient with personal history of acne and furuncles who was referred from a dermatology clinic with multiple abscesses, pustules and orifices that covered the parietal, temporal and occipital regions of the scalp with extension to the posterior cervical and beard area of the face (Figs. 1 and 2). When pressure was applied over the lesions, purulent drainage from the orifices was described. Bad odor and pruritus were present, but pain was absent. The lesions have started as a nodule around the vertex and have evolved in 5 years to cover most of the scalp. During this period, several antibiotics were used and local dressings were applied, under the suspicion of different diagnoses such as: S*taphylococcus* infection, *Candida* infection, folicullitis keloidalis, carbuncle, sycosis, folliculitis decalvans. No isotretinoin or biologic agents had been prescribed. One month prior to admission in our surgical clinic, the patient was treated with acitretin (30 mg daily PO, 30 days), penicilin G (1.500.000 IU/8 hour IV, 12 days), rifampicin (300 mg/12 hour PO, 14 days) and daily povidone-iodine wet to dry local dressings. All these therapeutic attempts were ineffective, the lesions progressed and the patient developed depression and social isolation.

The most severe complications of this disease are osteomyelitis and squamous cell carcinoma, which were excluded by MRI scan and puncture biopsy.^[2]^ Results of routine laboratory tests were within normal ranges, except for neutrophilic leukocytosis (17.9 × 10^**3**^/μl), mild normocytic normochromic anemia and elevated inflammatory markers (C-reactive protein: 122 mg/L, ESR: 75 mm/hour, fibrinogen: 816 mg/dl). Anti Streptolysin O and rheumatoid factor testing showed no pathological results. The serologic tests for syphilis, B and C hepatitis and HIV were negative. The coproparasitological test, pharyngeal and nasal exudates were also negative. A succession of bacteria were found to contaminate the lesions (*Streptococcus agalactiae, Enterococcus* spp, *Serratia odorifera*, *Serratia marcescens, Acinetobacter baumannii, Klebsiella* spp.) and IV antibiotics were administered according to the antibiogram in the following order: tigecycline 50 mg/12 hour for 21 days; amoxicilin/ clavulanate 1.5 g/8 hour along with gentamicin 80 mg/12 hour for 8 days and colistin sulfate 9,000,000 IU along with meropenem 3 g/24 hour for 14 days.

**Figure 3:**
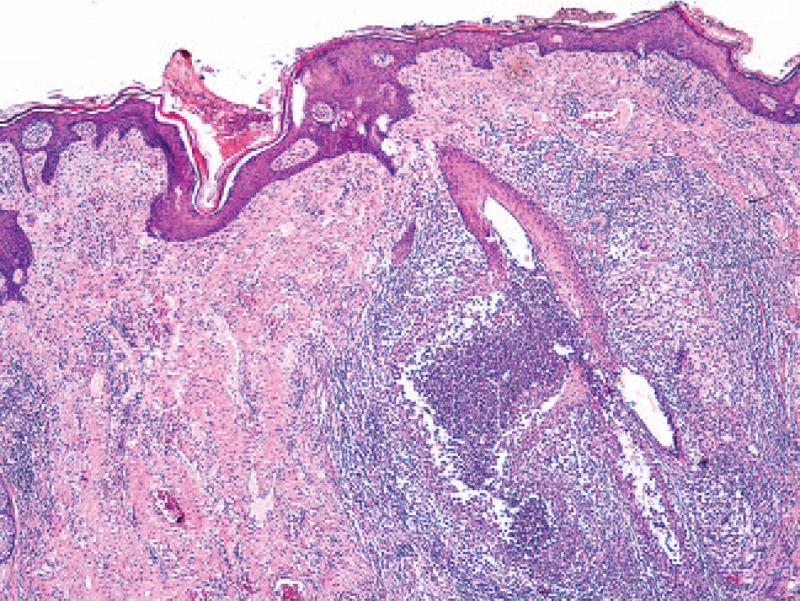
Folliculitis and perifolliculitis at the periphery with abscess formation (HE stain, ×10).

**Figure 4:**
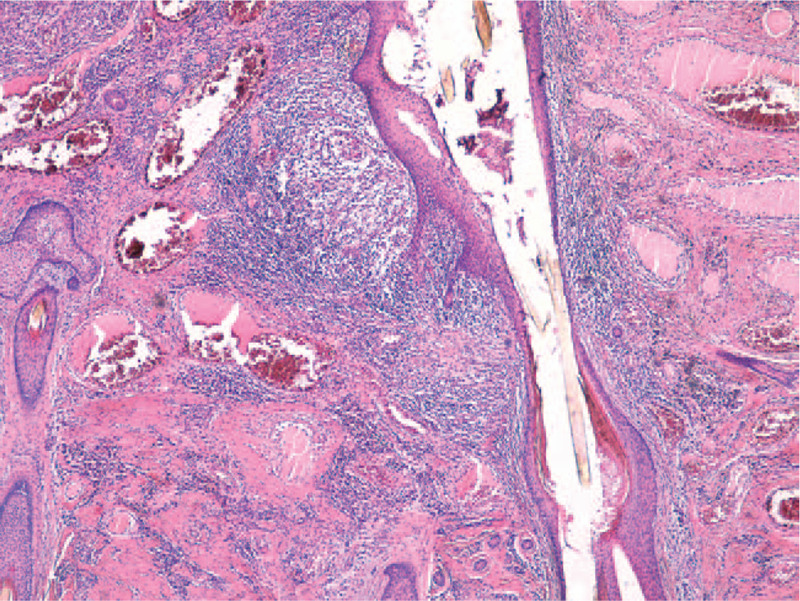
Hair follicles with dilatation of the follicular infundibulum and plugging, perifollicular chronic inflammation (HE stain, ×20).

**Figure 5:**
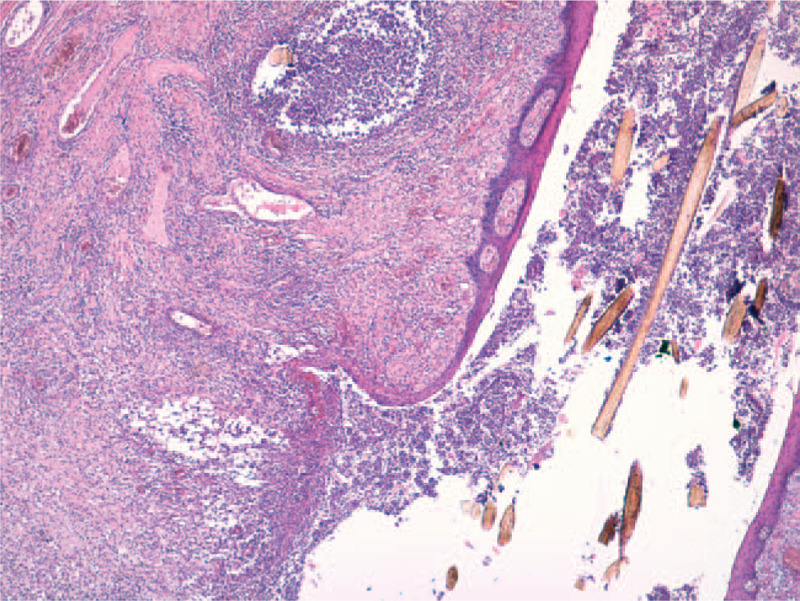
Free hair shafts in the dermis surrounded by foreign body giant cells (HE stain, ×20).

**Figure 6:**
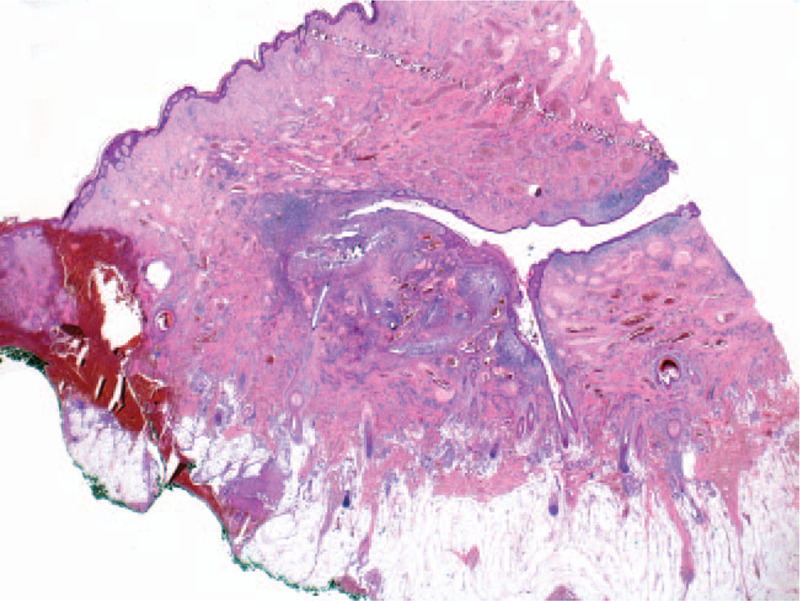
Multiple abscesses and sinus tracts (HE stain, ×4).

**Figure 7:**
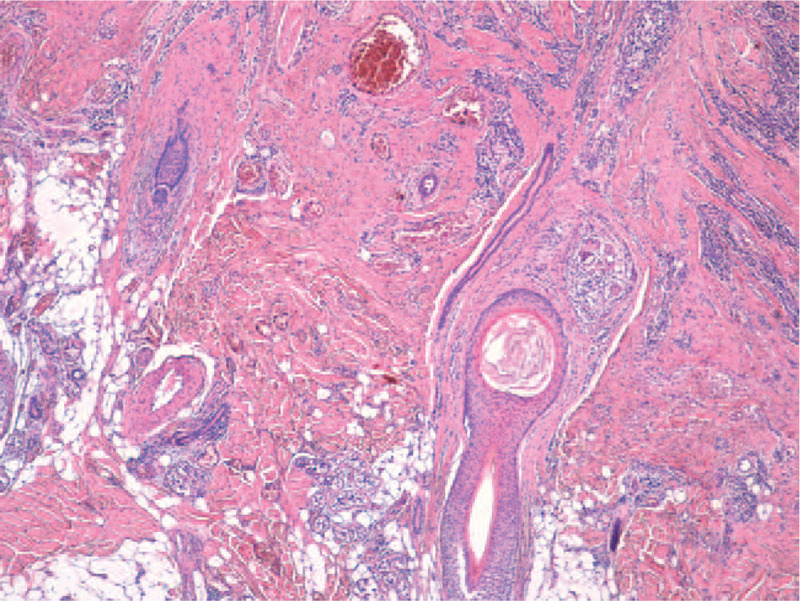
Fibrous tissue replacing the destroyed hair follicles (HE stain, ×20).

The histopathological exam confirms the clinical diagnosis of DCS. Lesions in different stages of evolution can be seen. Early histological changes of folliculitis and perifolliculitis at the periphery along with abscess formation can be found (Fig. 3). Hair follicles with dilatation of the follicular infundibulum and follicular clogging with heavy mixed inflammatory cell infiltrate (neutrophils, lymphocytes, plasma cells, histiocytes) are present centrally. Some hair follicles are destroyed and there are free hair shafts in the dermis. Focally, epithelioid and foreign body giant cells with formation of granulomata are present (Figs. 4 and 5). Advanced lesions such as multiple abscesses and sinus tracts, lined by stratified squamous epithelium are described (Fig. 6). Fibrous tissue replacing the destroyed hair follicles and sebaceous glands, elements that are representative for the final stages of the disease, complete the histopathological aspect (Fig. 7). Venous vascular proliferation has developed in the superficial dermis (possible reactive response) with absence of WT1 marker during immunohistochemistry.

**Figure 8:**
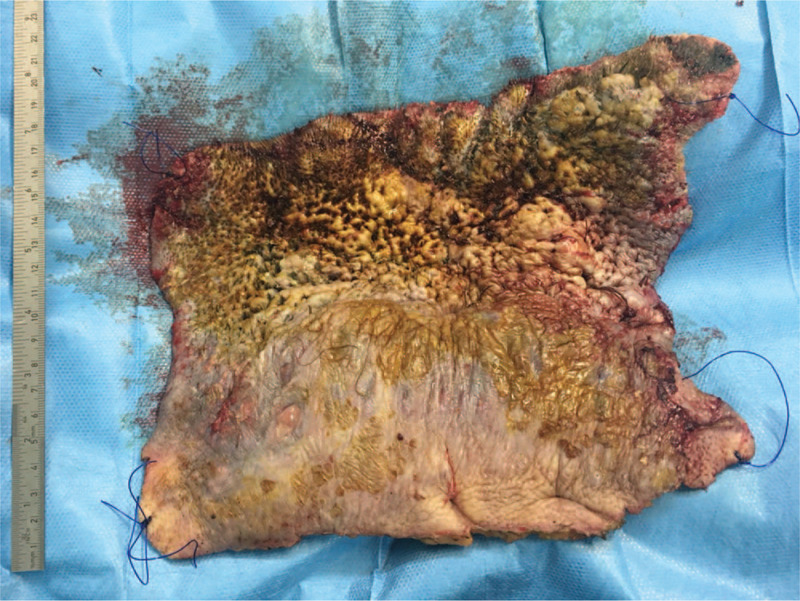
The excised scalp (17 × 25 cm).

To achieve eradication of the disease, surgical resection was performed just superficial to the galeal level as 1 stage procedure, with patient in prone position (Fig. 8). The intervention lasted 2 hours. The resulting defect measured approximately 650 cm.^2^ Postoperatively, wet to dry dressings with povidone-iodine and alternately, polyhexanide were applied twice daily.

**Figure 9:**
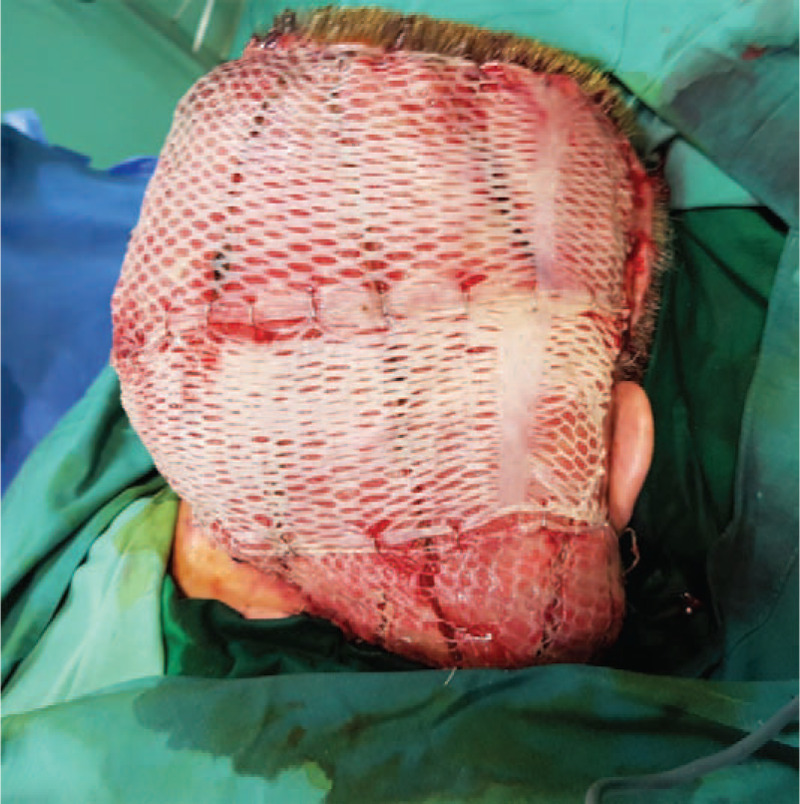
Scalp reconstruction with latissimus dorsi flap covered by meshed STSG.

Eight days after scalpectomy, reconstruction of the scalp using free muscular latissimus dorsi flap transfer, harvested from the right side was performed. End-to–end anastomosis of the thoracodorsal artery with the right superior thyroid artery and end-to-end anastomosis of the thoracodorsal vein with the anterior jugular vein were done. The intervention lasted 11 hours. The muscle flap dimension were 20 × 30 cm and it was covered by meshed STSG from the posterior thigh, another 8 days after, to ensure its viability (Fig. 9). Some of the graft failed (approximately 20%), so a regrafting had to be performed from the antero-lateral thigh and a tie-over dressing was applied over the scalp for 3 weeks. The patient developed seroma at the donor site that persisted for 1 month and was treated by needle aspiration.

Historical, current information and therapeutic interventions are summarized in the timeline (Table 1).

**Table 1:** Historical, current information, and therapeutic interventions.

	65-year-old Caucasian male patient with personal history of acne and furuncles			
Date (dd/mm/yy)	Summaries from Initial and Follow-up Visits	Diagnostic Testing (including dates)	Interventions	Medication
One month prior to addmission to Plastic Surgery Clinic	5-year history of multiple abscesses, pustules and orifices that covered the parietal, temporal and occipital regions of the scalp with extension to the posterior cervical and beard area of the faceBad odor and pruritusDepression and social isolation	- MRI scan- puncture biopsy- neutrophilic leukocytosismild normocytic normochromic anemia and elevated inflammatory markers- Anti Streptolysin O and rheumatoid factor: no pathological results. The serologic tests for syphilis, B and C hepatitis and HIV were negative. The coproparasitological test, pharyngeal and nasal exudates were also negative- Scalp wound culture*: Streptococcus agalactiae*		Acitretin (30 mg daily PO, 30 days),Penicilin G (1.500.000 IU/8h IV, 12 days), Rifampicin (300 mg/12h PO, 14 days) and daily povidone-iodine wet to dry local dressings
11/04/18Admission to Plastic Surgery Clinic	Multiple abscesses, pustules and orifices that covered the parietal, temporal and occipital regions of the scalp with extension to the posterior cervical and beard area of the faceBad odor and pruritusDepression and social isolation	Scalp wound culture *Streptococcus agalactiae*27/04/18 Scalp wound culture - *Enterococcus spp*08/05/18 Scalp wound culture - *Serratia odorifera*15/05/18 Scalp wound culture *- Serratia marcescens, Acinetobacter baumannii,*24/05/18 Scalp wound culture *- Klebsiella spp*04/06/18 Scalp wound culture –no growth07/06/18 Scalp wound culture - no growth18/05/18-The histopathological exam confirms the clinical diagnosis of DCS	16/04/18- surgical resection was performed just superficial to the galeal level24/04/18- reconstruction of the scalp using free muscular Latissimus Dorsi flap transfer03/05/18- The muscle flap was covered by meshed STSG from the posterior thigh25/05/18- regrafting had to be performed from the antero-lateral thigh and a tie-over dressing was applied over the scalp for 3 weeks	Tigecycline 50 mg/12h for 21 dayswet to dry dressings with povidone-iodine and alternately, polyhexanide were applied twice dailyAmoxicilin/ clavulanate 1,5 g/ 8h along with Gentamicin 80 mg/12h for 8 daysColistin sulfate 9,000,000 IU along with Meropenem 3g/24h for 14 days.
08/06/18 At discharge	Complete remission of symptoms and lesionsThe pustules that covered the beard area of the face also disappeared			
1-month follow up	Complete remission of symptoms and lesions along with a natural contour of the scalp		The patient developed seroma at the donor site that persisted for one month and was treated by needle aspiration	
18-month follow-up	Complete remission of the disease, including the facial lesions. The patient was grateful and very satisfied with the results, reported a high improvement of the quality of life and refused other means of enhancing the aspect of the scalp.No signs of seroma.			

**Figure 10:**
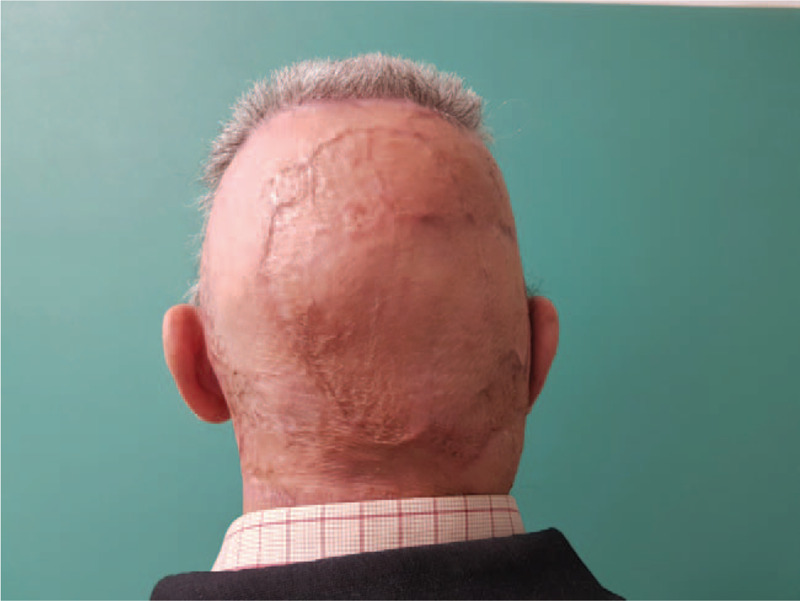
18 months postoperative aspect of the scalp.

**Figure 11:**
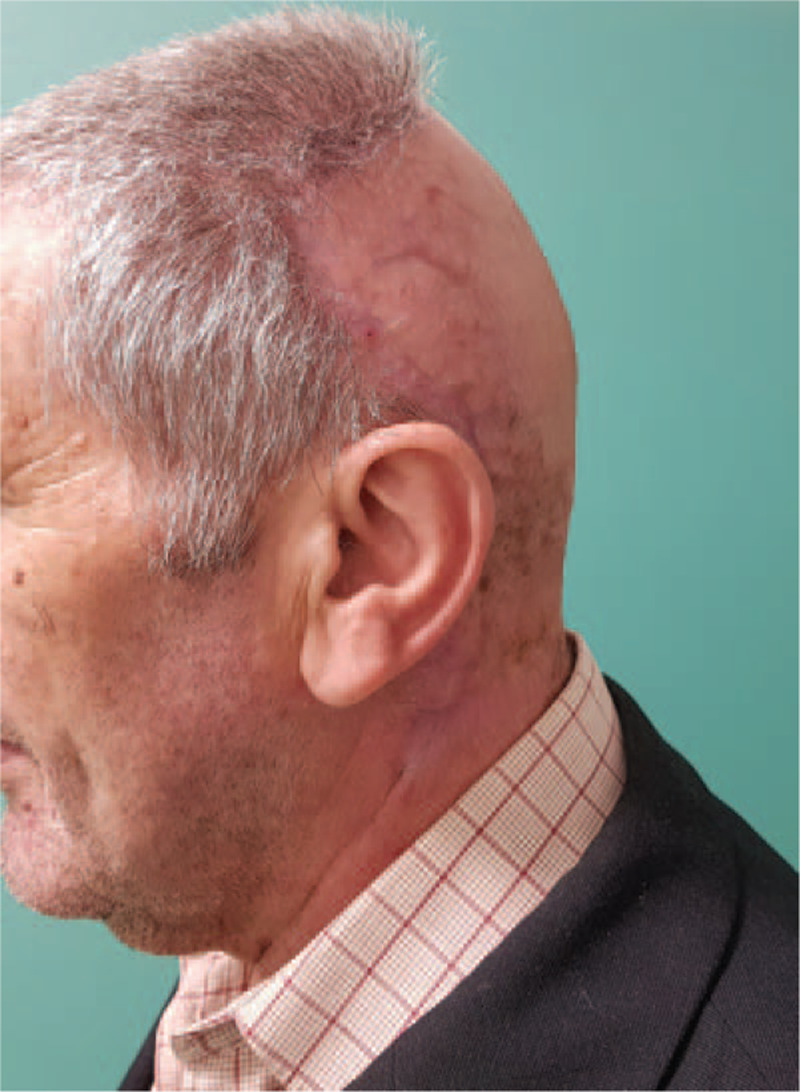
18 months postoperative aspect of the scalp.

Eighteen-month follow-up revealed a complete remission of symptoms and lesions along with a natural contour of the scalp (Figs. 10 and 11). The pustules that covered the beard area of the face, also disappeared.

## 2 Discussion

DCS is a rare disease that was first described by Spitzer. The etiology of DCS is unknown. Lesions at different stages may persist for years and cause considerable pain,^[4]^ but in our case it was absent. Bacterial infection of lesions is common, but it does not usually result in systemic symptoms.^[4]^ In our case, there were systemic symptoms.

Is this disease underdiagnosed? For our patient it took 5 years to be correctly diagnosed. Delays in diagnosis, similar to our case, have been reported in literature.^[3,5]^ An article conducted in Taiwan shows that in just 1 medical center were found more patients than in multiple medical centers in Europe. They raised the following questions: is this disease more common in Taiwan because of ethnic differences or is it underdiagnosed in Europe?^[6]^

Do we need a classification of the disease? In literature, different classifications of DCS can be found,^[6,7]^ but there are 2 main problems: first, they use different criteria. For example, Badaoui et al include pain,^[7]^ but Lee et al do not.^[6]^ Second, they correlate the same type of lesion with a different stage of the disease. For example, Branisteanu et al classify alopecia in early stages,^[3]^ while Lee et al only in advanced stages.^[6]^ In 2018, 2 dermatologists recommended that the therapy should be correlated with the stage of the disease.^[6]^ But how can we have a standardized treatment without a standard classification?

Do we know how to treat the disease? There is no consensus regarding the optimal medical management of DCS as the rarity of the disease results in data being limited to case reports and small case series. Some patients were treated well with antibiotics.^[3,8]^ Others recommend isotretinoin as the first line therapy,^[9,10]^ but frequent relapses after discontinuation are reported.^[7]^ Laser therapy and aminolevulinic acid-photodynamic therapy have also been tried with successful results.^[11,12,13]^ Most recently, there has been interest in the possible role that monoclonal antibodies can play in the treatment of the underlying inflammatory pathway of the disease. TNF inhibitors therapy is described.^[14,15]^ Clinical remission was achieved after treatment with adalimumab but relapse was observed following discontinuation of therapy.^[16]^ Mansouri et al consider that TNF inhibitors may be beneficial prior to surgical resection by reducing the extent of involvement, but they do not alter the structural disease and are not curative.^[14]^ Saireito, an herbal medicine was used in Japan with good results.^[17]^ Other articles report healing after zinc substitution therapy.^[18,19]^ As it can be seen, there are a lot of therapeutical approaches, but there is no standard treatment. If we correlate this with the fact that an early diagnosis is very difficult to establish (an issue often reported in literature),^[3,5]^ we have a possible explanation why patients end up in very advanced stages, where surgical procedure appears to be the only solution.^[20]^

When does surgery come into play? It is generally accepted that surgery is the recommended approach for nonresponsive cases. Lee et al recommend surgery to remove disfiguring hypertrophic scarring followed by hair restoration at a later stage, when disease activity is no longer observed.^[6]^ Hintze et al recommend that earlier resection may be considered due to significant psychosocial comorbidities associated with this disfiguring condition.^[2]^ It is also known that secondary squamous cell carcinoma arises after a long latency period within preexisting inflammatory and/or scarring lesions.^[21]^ An interesting aspect is that in cases where DCS was associated with spondyloarthritis, some patients reported improvement of arthritis after surgical therapy of the scalp.^[22]^ Prior reports and our own experience support that partial thickness scalp excision leads to cure of the disease. Scalp resection is performed to a level just deep to the disease, usually galeal or just subgaleal.^[2]^ To the best of our knowledge, there are no reported recurrences after scalpectomy.^[4,20,23,24,25]^There is only 1 case report where the patient was referred to a dermatologic surgeon for excision and after 1 year of remission the disease reappeared at the site of the surgical scar.^[26]^ Because conservative treatment of hidradenitis suppurativa, a related disease, cannot prevent recurrence, surgical treatment is the method of choice.^[27]^

Severe cases of DCS can lead to marked disfigurement, poor cosmetic appearance and bad odor.^[2,25]^ Prior reports and our own experience support that patients experience significantly improved quality of life following surgical resection and reconstruction of recalcitrant disease.^[2,4,23,24,25]^

We have chosen to reconstruct the scalp using free LD flap covered by skin grafts for several reasons: our scalp defect was large (about 650 cm^2^), therefore local flaps were not suitable. In the algorithms for scalp reconstruction proposed by Simunovic, it is stated that defects larger than 6 to 8 cm within the hair bearing scalp and 4 to 5 cm at the hairline require coverage with free flaps.^[28]^ Considering that muscle is useful for controlling infections due to its abundant vascularity,^[29,30,31]^ we decided that a free muscular transfer was suitable in our case because of the bacterial infection that contaminated the lesions. Taking into consideration the size of the defect, LD flap was used in our case because of its large dimensions and long constant pedicle. A main complaint regarding LD flap is the bulky appearance, but with time, it atrophies and approximates the normal contour of the scalp.^[32]^ Reconstruction with free flaps reduces the number of operations necessary to cover the defect, unlike serial tissue expansion or staged excision methods. Free flaps are also a good alternative when the patient does not have enough hair-bearing tissue to expand.^[33]^

Following scalpectomy, both STSG and FTSG are described in literature for scalp reconstruction as an alternative to free tissue transfer. Because the underlying calvarium provides a rigid infrastructure, that helps minimizing the effects of wound contraction, STSG are used more frequently than FTSG for scalp reconstruction.^[34]^ Some articles report covering the defect only with STSG with satisfactory aesthetic results.^[4,20,23,24,25]^ Wolff et al consider that STSG has been found to be less durable and its long-term reliability inferior to FTSG-when faced with injury caused by inadvertent trauma.^[35]^ In contrast, Schiavon et al describe skin grafting as a 2 dimensional technique that can lead to a disproportionately hollow reconstructed side compared to the nonoperated area.^[36]^ Even more, skin grafts applied on scalp defects are reported to produce a shiny nonmobile surface that is prone to ulceration.^[37]^

Regarding scalp reconstruction, hair transplantation is the final step in enhancing the appearance. It is worth mentioning that in advanced stages of the disease, the bald postoperative appearance is much more aesthetic than the preoperative one, even without hair restoration surgery.^[20]^ However, good aesthetic outcomes are reported after hair mini grafts transplantation on free LD flap.^[38]^ In our case, the patient was very satisfied with the final result and refused the hair transplant or any other means of improving the aspect of the scalp.

One other problem consists in whether it is recommended or not to practice hair restoration surgery in DCS, and if it is, when is the right time to do it. Risk of disease reactivation is believed to persist even in a burnt-out stage and after several years without treatment in inflammatory cicatricial alopecias. In addition, in 1 case of hair transplant for male pattern hair loss, folliculitis decalvans, a related disease, occurred 20 years after the procedure exclusively in the area of the hair grafts. Therefore, hair transplant surgery should be taken into consideration if the patient does not show any sign of reactivation of the disease, in the absence of any treatment.^[39]^ In our case, all the affected areas were completely excised and the scalp was reconstructed, using tissues with different histologic features, in order to reduce the chances of disease recurrence as much as possible.

An alternative to hair transplant is tissue expansion of the hair bearing surface of the scalp, an approach that can be used even if 50% or more of the scalp suffers from alopecia.^[40,41]^ Unfortunately, this method comes hand in hand with long periods of disfigurement, numerous clinical visits, and prolonged treatment.^[40]^ In addition, the reduce density of hair follicles that result after tissue expansion in a large scalp defect compromises the aesthetic appearance.^[34]^

To conclude, DCS is a rare and underdiagnosed disease. A worldwide accepted classification would facilitate a better understanding of this disorder and an earlier diagnosis. In addition, a standardized therapeutic approach, correlated with the classification, would have a strong positive impact on the long term management. Depression and social isolation should be taken into consideration in staging the severity of the disease and early surgical excision could be recommended when these 2 comorbidities severely alter the quality of life. Scalpectomy is an invasive but efficient treatment of dissecting cellulitis of the scalp and the best cost-effective coverage is obtained using microsurgical free transfer and meshed STSG, from our point of view. To the best of our knowledge, our patient represents the first case with DCS where free LD flap and meshed STSG was used to reconstruct the scalp after scalpectomy. Eighteen-month follow-up shows complete remission of the disease, including the facial lesions. The patient was grateful and very satisfied with the results, reported a high improvement of the quality of life and refused other means of enhancing the aspect of the scalp.

## Author contributions

**Conceptualization:** Zorin Petrişor Crăiniceanu, Nicoleta-Sara Baneu.

**Data curation:** Diana Szilagyi, Patricia Cristodor, Alexandru Pesecan.

**Methodology:** Vlad Adam Bloancă.

**Supervision:** Zorin Petrişor Crăiniceanu, Tiberiu Ioan Bratu.

**Writing – original draft:** Nicoleta-Sara Baneu.

## Abbreviations

Abbreviations: DCS = dissecting cellulitis of the scalp, FTSG = full thickness skin graft, LD = latissimus dorsi, MRI = magnetic resonance imaging, STSG = split thickness skin graft, TNF = tumor necrosis s factor, WT1 = Wilms tumor protein.
